# trieFinder: an efficient program for annotating Digital Gene Expression (DGE) tags

**DOI:** 10.1186/1471-2105-15-329

**Published:** 2014-10-13

**Authors:** Gabriel Renaud, Matthew C LaFave, Jin Liang, Tyra G Wolfsberg, Shawn M Burgess

**Affiliations:** Department of Evolutionary Genetics, Max Planck Institute for Evolutionary Anthropology, Leipzig, Saxony, D-04103 Germany; Computational and Statistical Genomics Branch, Division of Intramural Research, National Human Genome Research Institute, National Institutes of Health, Bethesda, MD 20892-8004 USA; Translational and Functional Genomics Branch, Division of Intramural Research, National Human Genome Research Institute, National Institutes of Health, Bethesda, MD 20892-8004 USA; Department of Biological Statistics and Computational Biology, Weil Institute for Cell and Molecular Biology, Ithaca, NY 14853-7202 USA

**Keywords:** RNA-Seq, Transcriptional profiling, DGE, SAGE

## Abstract

**Background:**

Quantification of a transcriptional profile is a useful way to evaluate the activity of a cell at a given point in time. Although RNA-Seq has revolutionized transcriptional profiling, the costs of RNA-Seq are still significantly higher than microarrays, and often the depth of data delivered from RNA-Seq is in excess of what is needed for simple transcript quantification. Digital Gene Expression (DGE) is a cost-effective, sequence-based approach for simple transcript quantification: by sequencing one read per molecule of RNA, this technique can be used to efficiently count transcripts while obviating the need for transcript-length normalization and reducing the total numbers of reads necessary for accurate quantification. Here, we present trieFinder, a program specifically designed to rapidly map, parse, and annotate DGE tags of various lengths against cDNA and/or genomic sequence databases.

**Results:**

The trieFinder algorithm maps DGE tags in a two-step process. First, it scans FASTA files of RefSeq, UniGene, and genomic DNA sequences to create a database of all tags that can be derived from a predefined restriction site. Next, it compares the experimental DGE tags to this tag database, taking advantage of the fact that the tags are stored as a prefix tree, or “trie”, which allows for linear-time searches for exact matches. DGE tags with mismatches are analyzed by recursive calls in the data structure. We find that, in terms of alignment speed, the mapping functionality of trieFinder compares favorably with Bowtie.

**Conclusions:**

trieFinder can quickly provide the user an annotation of the DGE tags from three sources simultaneously, simplifying transcript quantification and novel transcript detection, delivering the data in a simple parsed format, obviating the need to post-process the alignment results. trieFinder is available at http://research.nhgri.nih.gov/software/trieFinder/.

## Background

Interrogation of a transcriptional profile is a key component to understanding the biology of an organism at the molecular level [1–3]. By measuring the identity and abundance of RNA molecules at a given point in time, one can generate a snapshot of how the organism is responding to the environment. Accurate quantification of transcript abundance has therefore been the aim of techniques that have changed over the years with the advent of new technologies.

Serial analysis of gene expression, or SAGE, established the technique of using a single, consistent section of each RNA molecule to directly quantify transcript abundance [4]. Early SAGE required steps in which concatemerized cDNA fragments were cloned into a vector and sequenced. As such, SAGE fragments, or tags, were kept short (9–10 bp) as a means of maximizing the number of cDNA molecules that could be counted in a single vector insert.

Digital Gene Expression (DGE) is a concept first introduced after the realization that large scale sequencing of expressed sequences (e.g. EST projects) could give an indication of gene expression levels based on the frequency at which each gene sequence occurred in a data set [5]. The development of high-throughput sequencing paved the way for massively parallel signature sequencing, or MPSS, the first adoption of SAGE-type DGE using a high-throughput sequencing platform [6]. The general aim of MPSS – to directly quantify transcript abundance by counting tags – is similar to SAGE. Modifications of the approach, such as direct sequencing of individual cDNA fragments, make MPSS, and DGE in general, more amenable to scaling than traditional SAGE. MPSS was originally designed to produce relatively short tags (16–20 bp), partially in response to the short read lengths expected at the time. Even with short reads, the technique has proven useful in the assessment of gene expression [7, 8]. More recent iterations of the technology, such as the Ovation 3’-DGE System (NuGEN), have modified the protocol to produce longer tags. Rather than being defined by the reach of a type IIS restriction enzyme, modern DGE tags are limited only by read length and the distance of the main restriction site from the 3’ end of the transcript in question. We will hence use the term DGE when referring to this type of analysis.

Other technologies exist with which to examine the transcriptome. Microarrays are a well-standardized means of examining relative abundance for a defined set of transcripts [9]. RNA-Seq is an extremely flexible approach, and is an excellent means for detecting alternative splicing, exon boundaries, full-transcript sequence, and normalized transcript abundance [10–12]. However, DGE remains a well-suited and cost-effective approach to directly quantify transcript abundance counts within a given sample.

They key difference between transcript quantification by RNA-Seq and by DGE is the number of times a given transcript can be “hit”. In RNA-Seq, a single molecule of RNA can be hit multiple times, which necessitates normalization relative to transcript length in order to generate an estimate of the abundance of that transcript. For quantification, RNA-Seq hits after the first on a given molecule contribute no new information about the number of molecules of that transcript in the sample.

In contrast, that same molecule would be sequenced only once by DGE, because only the 3’-most fragment generated by the restriction enzyme is captured. The length of the total transcript has no effect on the capture of the DGE tag, meaning that raw read counts can be used to determine the representation of the transcript, without the need for additional normalization. In RNA-Seq, sequencing depth is correlated with transcript abundance; in DGE, sequencing depth is transcript abundance. No reads are superfluous, because each serves as the sole hit of a molecule of RNA. As a consequence, for an experiment with a given number of reads, DGE would be expected to have a better representation of rare transcripts than RNA-Seq. A corollary to this is that, if rare transcripts are of less interest for a given experiment, the number of reads generated could be reduced, thereby making a run of DGE a substantially less expensive option than RNA-Seq.

We have developed trieFinder to assist with the mapping and annotation of DGE tags generated from single-end high-throughput sequencing. The program generates a database of all possible DGE tags for a given restriction site, based on information from multiple sources, such as RefSeq, UniGene, and genomic DNA. The tag database is stored as a prefix tree, or “trie”, which can then be efficiently searched with the experimentally-generated tags. The program parses the results and generates a tab-delimited output detailing the hits to the various types of databases for a given tag. This parsed table gives the researcher flexibility in how the data can be analyzed and different priority schemes or subsets of the data can be used depending on the researcher’s needs. The user can then infer the biological relevance of each result by considering the number of mismatches and the inclusivity of the reference to which it mapped.

## Implementation

**Figure 1:**
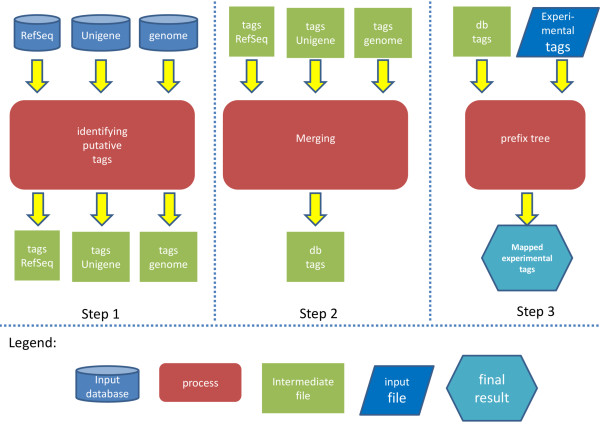
**Overview of the trieFinder workflow.** In step 1, each of the selected sequence databases, RefSeq, UniGene, and genome in this case, is scanned and all potential DGE tags in each database are identified. In the second step, these potential tags are merged into a single database of tags, db_tags. In step 3, the experimentally-determined tags are mapped to the db_tags database using a prefix tree. The net result is an annotation of each experimentally-determined tag with respect to the RefSeq, UniGene, and/or genome databases.

The software requires a set of experimentally sequenced tags, the sequence of the recognition site for the restriction enzyme used, and up to three target sequence databases. We recommend a conservative transcript database, like RefSeq; a liberal transcript database, like UniGene; and a genomic database. RefSeq is a source of curated, high-confidence transcripts, while UniGene will facilitate the analysis of low-abundance transcripts absent from RefSeq. The use of a genomic DNA database allows for the detection of unannotated transcripts. The first step in the trieFinder algorithm is to identify every putative tag in each target database (Figure 1). Since all tags stem from a restriction enzyme digestion, each tag will begin with a common prefix of usually four letters. Hence, the algorithm linearly scans the database to find every instance of the common prefix allowing for one mismatch. Allowing this mismatch helps identify restriction sites that exist in the genome, but which are not represented in the reference sequence due to polymorphisms. Upon finding a match, the nucleotides immediately downstream of the restriction site are stored in a temporary file. The length of this stored substring is defined by the user to be equal to the length of the experimental sequence tags. Since restriction enzyme sites are usually reverse palindromic (i.e. the reverse complement of the sequence is equal to the sequence itself, e.g. CATG), the database is scanned only on one strand. To account for tags derived from the reverse strand, the algorithm also stores the reverse complement of the nucleotides immediately upstream of the restriction enzyme site. Once the putative tags have been produced for every target database, the tags are sorted alphanumerically. A merging procedure finds identical tags from different target databases and merges them into a single contiguous file.

**Figure 2:**
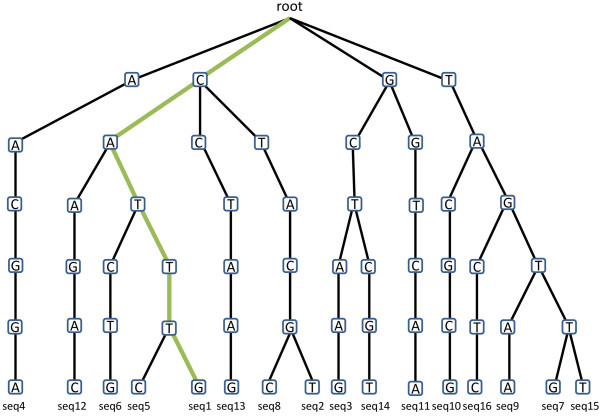
**An example of a prefix tree or “trie”.** Common prefixes are represented as common branches. Inexact searches for tags can be performed efficiently using recursive calls of the search algorithm until the user-defined maximum number of mismatches is met. To illustrate an exact search, the path leading to Seq1: CATTTG is highlighted in green.

The final step entails building a prefix tree (“trie”) using all of the potential tags (Figure 2). A prefix tree represents the common prefixes as a single path [13]. Apart from providing a memory-efficient representation of sequences sharing common prefix, exact searches can be handled in linear time with respect to the length of the input string. Searches allowing mismatches can be handled using recursive calls while keeping track of the mismatches found along the search path and stopping when the number of allowed mismatches is reached. Each sequence in a set of experimentally-determined tags is mapped by aligning it to putative tags using the prefix tree, allowing for a certain number of mismatches. The resulting hits are classified based on the database of origin of the putative tag, as well as the number of mismatches. Users can thus prioritize the reporting of the mapping data according to the inclusiveness of the sequence database (e.g. Refseq vs. UniGene) and the number of mismatches. So, for example, one might weigh a hit with one mismatch to RefSeq more heavily than a perfect hit to UniGene.

The main functional aspects of trieFinder were implemented in C++, while higher-level functions were implemented in Perl. We have tested the program in Linux, Mac OS X, and Cygwin environments. The memory requirements depend on the size of the input databases, but as an example, trieFinder required about 92 GB of disk space to store the indices of a 76 bp zebrafish index, and 10–20 GB of RAM to store the indices in memory.

## Results and discussion

### Functionality

A key feature that distinguishes trieFinder from other alignment software is that it is specifically tailored to the mapping of DGE tags. Rather than searching the total set of RNA or DNA sequences for possible alignments to the DGE tag, trieFinder creates a database, termed db_tags, which only contains putative sequence tags of a length defined by the user. In addition to reducing the search space, this feature ensures that the reported tag could have been produced by the restriction enzyme in question.

To accomplish this, trieFinder treats the fixed prefix sequence differently than the rest of the tag. This prefix represents the restriction site used to generate the tags, and is expected to be the first sequence detected on each tag. Regardless of the number of mismatches allowed in the full sequence, trieFinder tolerates no more than a single mismatch in the prefix. The motivation for allowing a single mismatch in the restriction site is to account for intraspecies single nucleotide polymorphisms where the sample would carry the correct recognition sequence but the reference would carry a mutation within this sequence. This is especially useful in species with highly polymorphic genomes, such as zebrafish [14].

The program is run from the UNIX command line. For a given set of DGE tags, the program has two main tasks: to build the db_tags database of all possible tags and to search db_tags for matches to experimental tags. These tasks can be accomplished with a single run of trieFinder, or db_tags can be built and searched separately. To create the db_tags database, the user runs the program by indicating the sequence of the fixed prefix of the tags, the location of local FASTA files for three nucleotide databases (such as RefSeq, UniGene, and genomic DNA), and the path to the desired location of db_tags. To search the db_tags database with experimentally-generated tags, the path to the input FASTQ file of DGE tags should be specified. An efficient means of presenting the data to trieFinder is to reduce identical reads into a single FASTQ entry, using the name field to store the number of reads represented by the sequence. Doing so avoids redundant mapping, and assists the user in interpreting the results. trieFinder will attempt to identify any relevant db_tags databases before creating any of them anew.

The search output is a tab-delimited file. Each line indicates the ID and sequence of a DGE tag, followed by the IDs of the exact and degenerate matches to each of the three nucleotide databases. Each of these potential matches is printed with a number to summarize how often a match was detected. Degenerate hits are those with at least one mismatch, up to a maximum defined by the user, with the caveat that no more than one mismatch is tolerated in the prefix for the reasons given above.

The RefSeq and UniGene results are the most directly applicable to transcript counting. The genomic DNA results are valuable for those tags with no hits in the transcript databases, as they will show the genomic location of potential novel transcripts.

### Limitations

Care must be taken when preparing DGE reads to present to trieFinder, especially when dealing with transcripts in which the distance from the restriction site to the end of the sequence is less than the length of the read. In these relatively rare cases, trimming of the DGE tags and modification of the database parameters are necessary to avoid false negatives. For example, if the DGE tags are all 76 bp in length, but the restriction site in a given transcript is only 30 bp away from its end, the DGE tag will include about 46 bp worth of poly-A and adapter sequence that will prevent mapping unless it is trimmed prior to running trieFinder. Users may find programs like cutadapt to be useful for this purpose [15].

In addition, the parameters with which the db_tags database is built need to be slightly modified to recover such a tag. If trieFinder were instructed to create a database of all plausible tags of 76 bp, it would not create tags for which the restriction site were less than 76 bp from the end of the FASTA sequence entry. Because of this, the correctly-trimmed read from the sample above would need to be mapped to a shorter database – in this case, a database built for 30 bp tags.

It is worth noting that reads shorter than the total length of the database fragments can be mapped, a benefit of storing the database information in a trie. For example, if a database were built for 100 bp tags but sequencing were only done to 76 bp, trieFinder would still be able to map most of the reads. However, in that scenario, tags generated from restriction sites less than 100 bp from the end of the transcript would be missed; retrieving them would require a database built for shorter fragments.

### Comparisons to existing software

To our knowledge, there are no algorithms or software designed specifically to provide both mapping and annotation of DGE tags. However, we found that the mapping functionality of trieFinder^a^ compares favorably with the short-read aligner, Bowtie^b^ [16]. It should be noted that this comparison is only possible when allowing no more than one mismatch overall. Otherwise, trieFinder is the only program of the two that can treat the number of mismatches in the common 4-bp prefix differently from those in the rest of the read.

Both programs were used to analyze 327,240 unique 20-bp DGE reads from zebrafish [8]. Sequence files from the same three sources that were used to create the db_tags databases (RefSeq, UniGene, and genomic sequence) were indexed as Bowtie target databases. We ran Bowtie on the three indexes separately, using settings that aligned about as many reads as trieFinder (Table 1). trieFinder was consistently faster than Bowtie in both overall CPU and wallclock time. In addition, the trieFinder output allows the user to examine the results from all three databases simultaneously, without needing to switch between multiple files. The trieFinder output also automatically prints out the number of entries matched by the read, which can be useful in determining the reliability of a match rather than having to parse multiple alignment files to summarize the alignment results. While trieFinder is faster than Bowtie, its key advantage is that the parsed output makes it easier for the user to prioritize mappings and interpret the data.

**Table 1:** **Performance of trieFinder versus Bowtie**

	Mean CPU time	CPU time SD	Mean wallclock time	Wallclock time SD	Peak virtual memory (MB)	Reads aligned (%)
trieFinder	3:00:53	0:00:35	2:24:17	0:00:33	> 4096	84.55
Bowtie	3:46:40	0:03:41	3:47:49	0:03:36	2564	84.51

Performance of trieFinder (commit aca5281183) and Bowtie (version 1.0.0, commit fe7a830e31; 64-bit) when aligning 327,240 unique 20-bp DGE reads from zebrafish, allowing a single mismatch. trieFinder was run using default settings. To match trieFinder as closely as possible, Bowtie-build was used to build three indexes based on the three database files (RefSeq, UniGene, and genomic) with default settings. The UniGene input was modified to remove comment lines inconsistent with the FASTA format when building the Bowtie indexes. Three Bowtie alignments were run, one for each index, using the settings ‘-v 1 -a -y -t --fullref --sam --sam-nohead’. The times represent the mean of the sum of all steps, including database/index creation and the sequence search, for three runs of each program. All runs were performed on a server with 64 GB of RAM and eight 800 MHz Quad-Core AMD Opteron processors. Times are displayed as hours:minutes:seconds. DGE reads are from Liang *et al*., 2012 [8]. CPU, central processing unit; SD, standard deviation.

## Conclusions

trieFinder provides an efficient means to map and annotate sequence tags that are generated by DGE, a cost-effective sequencing technique for direct transcript quantification. Our use of a trie data structure allows for rapid, accurate searches of sequence databases. trieFinder compares favorably with existing software, and produces an output format amenable to transcript quantification.

## Availability and requirements

- **Project name:** trieFinder
- **Project home page:**http://research.nhgri.nih.gov/software/trieFinder/
- **Operating system:** UNIX
- **Programming languages:** C++, Perl
- **Other requirements:** zlib; FASTA files from RefSeq, UniGene, and the genomic DNA sequence
- **License: GPLv3**
- **Any restrictions to use by non-academics:** None

## Endnotes

^a^Commit aca5281183d4825111dab74d319c5cc61be4309a.

^b^Commit fe7a830e31e467d9f57beab798100d0c941ee0ee.

## Abbreviations

DGE: Digital Gene Expression
MPSS: Massively parallel signature sequencing
SAGE: Sequential analysis of gene expression.
